# Beneficial effects of intrapulmonary percussive ventilation in patients with respiratory insufficiency in the ICU

**DOI:** 10.1186/cc12057

**Published:** 2013-03-19

**Authors:** I Blum, R Janssen-Dean, A Overdijk van, B Speelberg

**Affiliations:** 1 St Anna Hospital Geldrop, Geldrop, the Netherlands

## Introduction

Intrapulmonary percussive ventilation (IPV) is a therapy that is used to clear endobronchial secretions. The IPV ventilator was designed and developed by FM Bird in 1979. The ventilator consists of a phasitron that delivers rapid, high-flow, mini bursts of oxygen, mixed with air. The potential aims of this mechanism are pulmonary recruitment, improved mucus clearance with a direct high-frequency oscillatory effect. We investigated whether IPV has a positive effect on ventilatory values in adult patients on the ICU.

## Methods

All patients presenting during a 4-month period in 2011 with respiratory insufficiency on our mixed adult ICU were included in this study. Patients were monitored before, directly after and 15 minutes after therapy with IPV using a Bird Intrapulmonary Ventilator Model IPV-2C. All patients received IPV for a period of 20 minutes consisting of two cycles of 10 minutes. Peripheral oxygen saturation (SpO_2_), tidal volume (Vt), respiratory rate, end-tidal CO_2 _(ET-CO_2_), dynamic lung compliance (C-dyn) and work of breathing (WOB) were monitored at the different time points. Paired Student *t *tests were performed in order to compare the values immediately before IPV, with directly after therapy and 15 minutes later. *P <*0.05 was considered significant.

## Results

Eighty-three patients were examined. SpO_2 _improved significantly from 93.7 ± 3.7 before IPV to 95.7 ± 2.8 after IPV (*P <*0.001) and 15 minutes later to 95.2 ± 2.8 (*P <*0.001). Vt improved from 418 ± 111 before IPV to 476 ± 102 directly after (*P <*0.001) and to 480 ± 131 15 minutes later (*P <*0.01). Respiratory rate improved from 24 ± 6 to 23 ± 6 only after 15 minutes significantly (*P <*0.01). WOB and C-dyn did not change. ET-CO_2 _decreased from 34.9 ± 14.8 to 33.3 ± 13.3 (*P <*0.05) directly after IPV and to 32.1 ± 12.8 (*P <*0.01) 15 minutes later.

## Conclusion

In this study we demonstrated a beneficial effect of IPV on oxygen saturation, tidal volume and end-tidal CO_2_. IPV has this effect in addition to the mobilization of bronchial secretions.
